# Supplementary material for: A divide-and-conquer approach based on deep learning for long RNA secondary structure prediction: Focus on pseudoknots identification
Source: PLoS One. 2025 Apr 25;20(4):e0314837. doi: 10.1371/journal.pone.0314837 (PMC12026937; doi:10.1371/journal.pone.0314837)
Supplement: S1 File — The implementation details of DivideFold are given, along with detailed results for pseudoknot prediction and secondary structure prediction including pseudoknots of DivideFold, IPknot [16,17], ProbKnot [15], KnotFold [33], pKiss [8,9] and UFold [27]. (PDF) [file pone.0314837.s001.pdf]

A divide-and-conquer approach based on deep learning for long RNA secondary structure prediction: focus on pseudoknots identification - supplementary file

Loïc Omnes[1,2], Eric Angel[1], Pierre Bartet[2], François Radvanyi[3], Fariza Tahi[1*],

1 Université Paris-Saclay, Univ Evry, IBISC, 91020 Evry-Courcouronnes, France.
2 ADLIN Science, 91037 Evry-Courcouronnes, France
3 Molecular Oncology UMR144, CNRS - Institut Curie, 75005 Paris, France

* fariza.tahi@univ-evry.fr

Evaluation of structure prediction models on bpRNA-NF-15.0

We evaluate the different benchmarked methods (IPknot, ProbKnot, KnotFold, pKiss and UFold) to decide which one to use as the structure prediction model in our approach on bpRNA-NF-15.0.

Their performances and computation times are shown in Fig 1. UFold only accepts sequences shorter than 600 nt. On bpRNA-NF-15.0, KnotFold and IPknot provide similar performances, better than that of ProbKnot, pKiss and UFold. We choose to use KnotFold as the structure prediction model in our approach on bpRNA-NF-15.0.

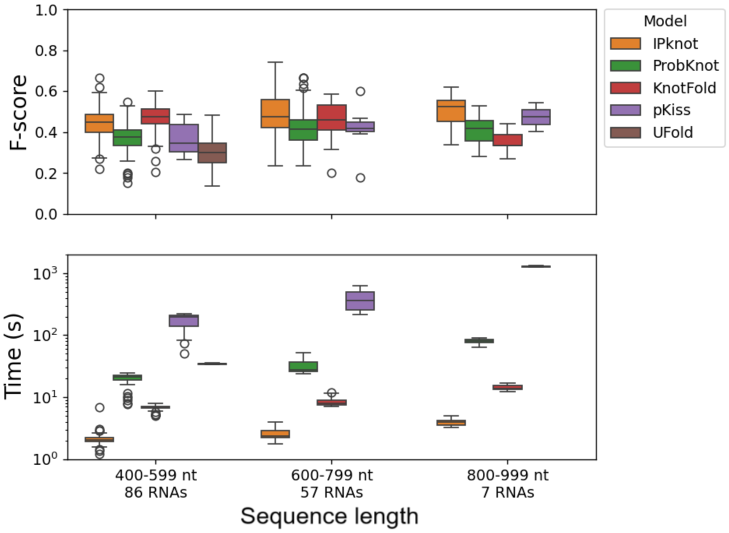

Fig 1. Structure prediction models evaluation by sequence length on bpRNA-NF-15.0. The F-score for secondary structure prediction including pseudoknots depending on the input sequence length is shown for IPknot [1, 2], ProbKnot [3], KnotFold [4], pKiss [5, 6] and UFold [7] on bpRNA-NF-15.0. The computation times are displayed for reference in a log scale.

Maximum fragment length hyperparameter on bpRNA-NF-15.0

We evaluate our approach for different values of the maximum fragment length up to 500 nt on bpRNA-NF-15.0, for the same reasons stated in the manuscript. To ensure that the results

are compared on the same data, we only consider here sequences longer than 500 nt.

We display in Fig 2 the mean compression rate and break rate of our divide model for different values of the maximum fragment length. The F-score for secondary structure prediction is also indicated for reference. On bpRNA-NF-15.0, we can see that the break rate is significantly higher. The F-score increases steeply with the maximum fragment length, suggesting that the model would likely benefit from higher values for the maximum fragment length. Unfortunately, the sequences in bpRNA-NF-15.0 are too short for this to be possible.

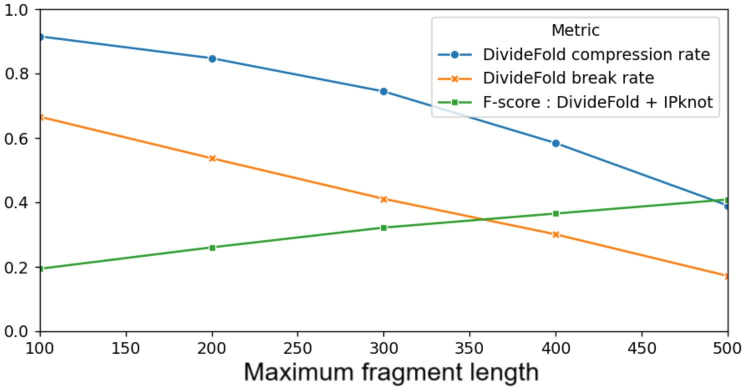

Fig 2. Compression and break rates with respect to maximum fragment length on bpRNA-NF-15.0. Mean compression rate and break rate of our divide model for different values of the maximum fragment length hyperparameter, for sequences longer than 500 nucleotides on bpRNA-NF-15.0. The F-score for secondary structure prediction is shown for reference.

We measure the F-score for the secondary structure prediction including pseudoknots and we separate the sequences from bpRNA-NF-15.0 into groups according to their lengths. We show the results in Fig 3 for bpRNA-NF-15.0. It is confirmed that the model would most likely benefit from higher values for the maximum fragment length. However, this is impossible since the sequences in bpRNA-NF-15.0 are too short, and we have to settle for 500 nt for the maximum fragment length on bpRNA-NF-15.0.

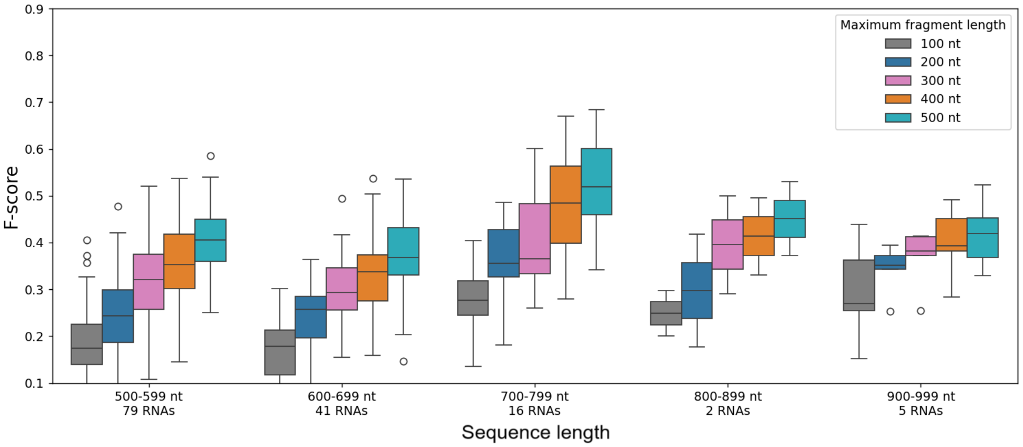

Fig 3. Secondary structure F-score for DivideFold with respect to maximum fragment length, by sequence length, on bpRNA-NF-15.0. The F-score for secondary structure prediction including pseudoknots depending on the input sequence length for RNAs longer than 500 nt is shown for DivideFold, using KnotFold as the structure prediction model, for different values for the maximum fragment length, on bpRNA-NF-15.0.

We choose a value of 500 nucleotides for the maximum length of a fragment in our approach on bpRNA-NF-15.0, and we focus on longer RNAs.

Pseudoknot prediction results on bpRNA-NF-15.0

The pseudoknot prediction performance of DivideFold on bpRNA-NF-15.0 is evaluated here. DivideFold is compared to IPknot [1,2], ProbKnot [3], KnotFold [4], pKiss [5,6] and UFold [7] on bpRNA-NF-15.0 for sequences longer than 500 nt. UFold is unable to predict RNAs longer than 600 nt.

We show the mean pseudoknot prediction results in Table 1 for bpRNA-NF-15.0. IPknot, ProbKnot and pKiss show poor results when predicting pseudoknots on bpRNA-NF-15.0. KnotFold performs much better and manages to find 70.0% of the pseudoknots on average on bpRNA-NF-15.0. However, it also predicts many false positives, leading to a low precision. In contrast, our approach finds less pseudoknots than KnotFold on average on bpRNA-NF-15.0, but has a better precision, ultimately leading to a higher F-score.

Table 1. Pseudoknot prediction performance on bpRNA-NF-15.0.

Model	Recall	Precision	F-score
DivideFold + KnotFold	0.509	**0.114**	**0.178**
KnotFold	**0.700**	0.071	0.128
pKiss	0.145	0.073	0.088
IPknot	0.036	0.066	0.042
ProbKnot	0.007	0.018	0.009

The performance of DivideFold, IPknot [1,2], ProbKnot [3], KnotFold [4] and pKiss [5,6] is reported here for RNAs longer than 500 nt on bpRNA-NF-15.0.

The pseudoknot prediction performance depending on the RNA sequence length is also shown in Fig 4 for bpRNA-NF-15.0. DivideFold reaches a higher F-score than other benchmarked tools in most cases.

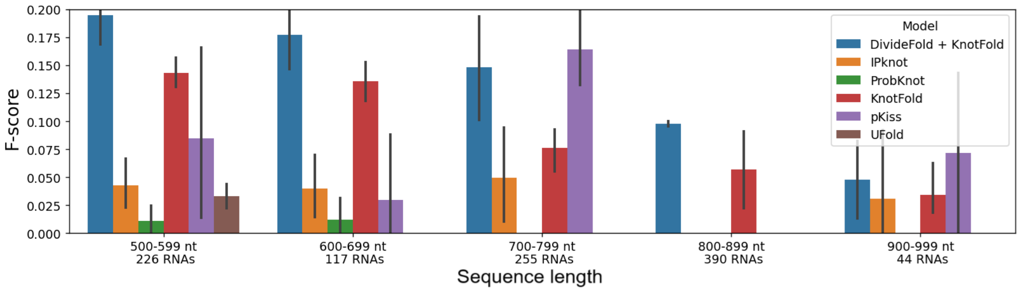

Fig 4. Pseudoknot prediction performance by sequence length on bpRNA-NF-15.0. The F-score is shown for pseudoknot prediction depending on the input sequence length for RNAs longer than 500 nt on bpRNA-NF-15.0. Confidence intervals are shown in the black bars. The performance of DivideFold, IPknot [1,2], ProbKnot [3], KnotFold [4], pKiss [5,6] and UFold [7] is reported here. Missing bars mean that the performance is very close to zero and cannot be seen.

Secondary structure prediction results including pseudoknots on bpRNA-NF-15.0

We evaluate here the secondary structure prediction performance including pseudoknots on bpRNA-NF-15.0. We also compare DivideFold to IPknot [1,2], ProbKnot [3] and KnotFold [4], pKiss [5,6] and UFold [7], on bpRNA-NF-15.0 for sequences longer than 500 nt. As before, UFold does not accept sequences longer than 600 nt. We show the results in Table 2 for bpRNA-NF-15.0. On bpRNA-NF-15.0, DivideFold yields slightly worse secondary structure F-score including pseudoknots compared to KnotFold and IPknot, showing that the generalization to unseen families is still a challenge for RNAs shorter than 1,000 nt. Nonetheless, all the benchmarked tools yield rather low F-scores on bpRNA-NF-15.0, and none them are able to reach satisfactory performance.

Table 2. Secondary structure prediction performance including pseudoknots on bpRNA-NF-15.0.

Model	Recall	Precision	F-score
DivideFold + KnotFold	0.443	0.418	0.424
KnotFold	**0.581**	0.383	0.461
pKiss	0.493	0.334	0.395
IPknot	0.483	**0.457**	**0.464**
ProbKnot	0.492	0.336	0.398

The performance of DivideFold, IPknot [1,2], ProbKnot [3], KnotFold [4] and pKiss [5,6] is reported here for RNAs longer than 500 nt on bpRNA-NF-15.0.

We also display the secondary structure prediction performance including pseudoknots depending on the RNA sequence length on bpRNA-NF-15.0 in Fig 5. On bpRNA-NF-15.0, DivideFold tends to yield weaker results than the other methods.

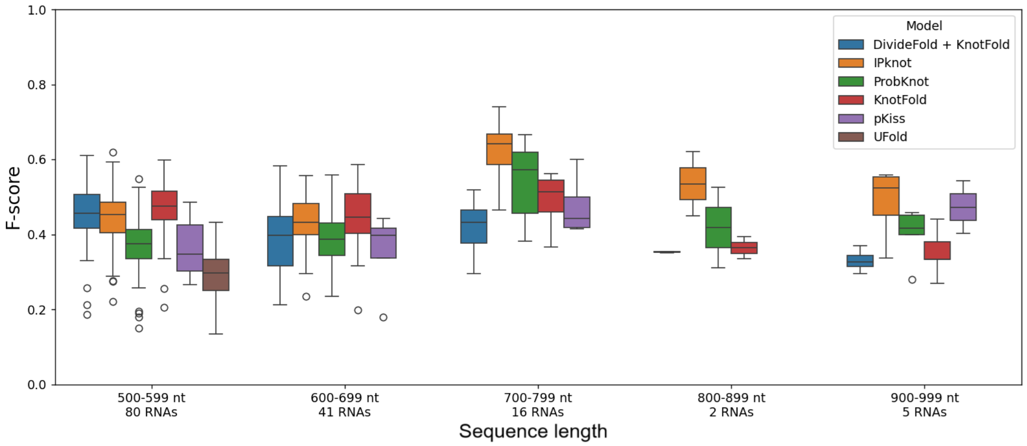

Fig 5. Secondary structure prediction performance including pseudoknots by sequence length on bpRNA-NF-15.0. The F-score is shown depending on the input sequence length for RNAs longer than 500 nt on bpRNA-NF-15.0. The performance of DivideFold, IPknot [1,2], ProbKnot [3], KnotFold [4], pKiss [5,6] and UFold [7] is reported here.

References

1. Sato K, Kato Y, Hamada M, et al. IPknot: Fast and Accurate Prediction of RNA Secondary Structures with Pseudoknots Using Integer Programming. Bioinformatics. 2011;27(13):i85–i93. doi:10.1093/bioinformatics/btr215.

2. Sato K, Kato Y. Prediction of RNA Secondary Structure Including Pseudoknots for Long Sequences. Briefings in Bioinformatics. 2022;23(1):bbab395. doi:10.1093/bib/bbab395.

3. Bellaousov S, Mathews DH. ProbKnot: Fast Prediction of RNA Secondary Structure Including Pseudoknots. RNA. 2010;16(10):1870–1880. doi:10.1261/rna.2125310.

4. Gong T, Ju F, Bu D. Accurate Prediction of RNA Secondary Structure Including Pseudoknots through Solving Minimum-Cost Flow with Learned Potentials. Communications Biology. 2024;7(1):1–13. doi:10.1038/s42003-024-05952-w.

5. Theis C, Janssen S, Giegerich R. Prediction of RNA Secondary Structure Including Kissing Hairpin Motifs. In: Moulton V, Singh M, editors. Algorithms in Bioinformatics. Berlin, Heidelberg: Springer; 2010. p. 52–64.

6. Janssen S, Giegerich R. The RNA Shapes Studio. Bioinformatics. 2015;31(3):423–425. doi:10.1093/bioinformatics/btu649.

7. Fu L, Cao Y, Wu J, et al. UFold: Fast and Accurate RNA Secondary Structure Prediction with Deep Learning. Nucleic Acids Research. 2022;50(3):e14. doi:10.1093/nar/gkab1074.
