# Supplementary material for: A divide-and-conquer approach based on deep learning for long RNA secondary structure prediction: Focus on pseudoknots identification
Source: PLoS One. 2025 Apr 25;20(4):e0314837. doi: 10.1371/journal.pone.0314837 (PMC12026937; doi:10.1371/journal.pone.0314837)
Supplement: Attachment — Submitted filename: Response to Reviewers.pdf [file pone.0314837.s002.pdf]

We thank the reviewers for their valuable comments.
Answers are in red.

Reviewer #1: This paper proposes a novel divide-and-conquer method, DivideFold, to address the challenges of predicting RNA secondary structures, particularly pseudoknots, in long sequences. DivideFold recursively partitions RNA sequences into smaller fragments, applies existing structure prediction models to each fragment, and reassembles the overall structure. The method demonstrates superior performance in pseudoknot prediction compared to existing models, with significant computational efficiency for long RNA sequences. Furthermore, the authors provide open access to the DivideFold source code and datasets, enhancing the reproducibility of the research.

Comment : The paper splits the dataset based on sequence similarity, but this approach poses a risk of overfitting. RNA sequences within the same family often share characteristic structural patterns, and splitting solely based on sequence similarity can lead to an overlap between the training and test sets, artificially inflating the model's performance.
To address this issue, it is recommended to split the data based on RNA families. For instance, if bpRNA_1m is used for training, an independent dataset such as bpRNAnew dataset (Sato et al., 2021) should be used for testing. This approach would provide a more realistic evaluation of the model's generalization capabilities, particularly for unseen RNA families.

Answer : We originally intended to check for generalization to unseen families. Unfortunately, bpRNA-new only contains sequences shorter than 500 nt, which makes it unusable in our approach. We added a comment on **page 8** to that effect.
However, splitting data based on a 80% sequence similarity threshold with the CD-HIT-EST software is a common practice in machine learning tools for RNA secondary structure prediction (SPOT-RNA, Singh et al., 2019 / MXfold2, Sato et al., 2021 / SPOT-RNA2, Singh et al., 2021 / UFold, Fu et al., 2022 / CNNFold, Saman Booy et al., 2022 / KnotFold, Gong et al., 2024).

Comment : In DivideFold, detecting cut points for splitting sequences into inner and outer fragments requires predicting two cut points simultaneously. This necessitates evaluating the relationships between all pairs of nucleotides, leading to a computational complexity of at least O(n^2).
The paper claims that the cut point detection algorithm operates with O(n) complexity, but this seems to omit the simultaneous prediction of inner and outer cut points or rely on simplifications. A more detailed explanation of the algorithm and its computational complexity is necessary for clarity and validation.

Answer : The divide model indeed operates in a O(n) time complexity (aside from the motifs insertion step, as stated in the manuscript, but this step is very fast compared to the others in practice). This is because the divide model does not evaluate the relationships between all pairs, but instead is based on a deep one-dimensional convolutional network, granting it a O(n) time complexity by construction (see Fig. 6). Through the use of decreasing dilation rates, the model is able to capture long-range relationships in the sequence.
For example, at a kernel size 3, at a position i in the sequence, the first layer of dilation rate 256 will compute features based on the three nucleotides at positions i-256, i, and i+256,

rather than all the nucleotides in the [i-256, i+256] range.
The second layer of dilation rate 128 will compute features based on the three nucleotides at positions i-128, i, and i+128. This goes on for successive layers, meaning that exactly three nucleotides are used for computation at each position in the sequence and at each layer. As a result, the computation time grows in linear time in regards to the sequence length.
This helps give the model a sense of the long-range relationships in the sequence, mimicking the behavior of a U-Net architecture, while avoiding the need to evaluate every possible pair in the sequence.
We added a comment on **page 3** for clarification.

Comment : The paper describes the removal of pseudoknots before cut point selection, but it does not clarify how the nucleotide pairs involved in pseudoknots ((i, j) and (k, l) for i-
Minimizing free energy for thermodynamic stability,
- Prioritizing longer or more stable stems,
- Heuristic approaches to preserve the overall structure.

The criteria for removal should be clearly explained, because which base pairs to remove would affect the accuracy of the prediction.

Answer : We used the PyCogent project to remove pseudoknots, which offers functions to do this based on six different possible criteria:
- dynamic programming, minimizing free energy
- prioritizing longer stems
- prioritizing long-range stems
- prioritizing stems from left to right (or right to left)
- conflict elimination, prioritizing regions with the most conflicts
- conflict elimination, prioritizing regions with the most (conflicts **-** base pairs)

Until now, we were using the criterion to prioritize longer stems in our study.
However, following your comment, we studied the effect of choosing any of these 6 criteria for pseudoknot removal before training. We re-trained our model with the different criteria and observed an increase in performance when prioritizing **long-range** stems rather than longer stems during pseudoknot removal prior to training. This could be because ensuring that the long-range stems are conserved would yield more stable and accurate cut point training labels when generating them at ideal positions (i.e in between the different stems that are minimally nested, i.e the long-range stems). Because of this, we choose to use the long-range stems criterion.
We added a comment on **page 6** for clarification.
We also updated the performance of our approach in the figures and tables based on the new performance observed under the “long-range stems” criterion.

Reviewer #2: The accurate prediction of RNA secondary structures is still a great challenge, especially for RNAs with long sequence and/or pseudoknots. This work proposed a novel approach to solve this problem by partitioning a long sequence into shorter ones. For this approach, the key is how to maintain the stems formed by long-range bases. The most novel point of this work is proposing a method to ensure that the long-range pairing is not disrupted by the partition. The benchmarks show that in most cases their method overperforms the existing methods in prediction of secondary structure and pseudoknots for long RNAs, including computation time. The results may also be important to understand the

mechanism of RNA secondary structures. This paper is clearly written too. I recommend it for publication after minor revisions.

Comment : 1. Line 151: “For each motif, we check for matches in the sequence through regular expressions and count the number of possible configurations for the motif to be inserted at each point in the sequence.” Give details using a picture

Answer : Here is a picture that will hopefully clarify the motifs insertion process:

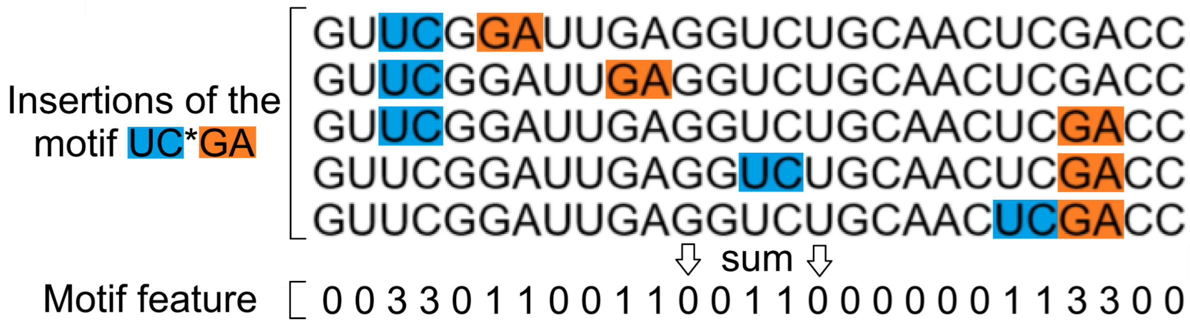

For a given RNA sequence, the possible configurations of a motif are found inside the sequence using regular expressions. Then, at each nucleotide in the sequence, we sum the number of configurations that happen at that position.
This is repeated for each motif to be inserted, each time resulting in a different feature for the sequence.
We added this figure to the paper on **page 5**, and clarified the process of motif insertion in its caption.

Comment : 2. It is better to give the average performance of cut-point prediction because this is the key to this approach.

Answer : For this reason, we have provided two metrics of interest for evaluating the quality of the partition: the break rate and compression rate. These are detailed in the manuscript on **page 9** and showcase two important properties of the partition:
- how much of the structure it is able to conserve
- by how much the fragments are shortened compared to the original sequence.

Comment : 3. It is interesting to know how about the performance of this approach for middle length, e.g., 500-1000nt. Suggest giving a brief discussion.

Answer : We added a new subsection on **pages 13-14** detailing the performance of DivideFold for RNAs between 500 nt and 1000 nt long.
DivideFold is able to give better results than KnotFold regarding the F-score in pseudoknot prediction, but not for structure prediction including pseudoknots.
We conclude that DivideFold is better suited for RNAs longer than 1,000 nt, which become increasingly difficult to deal with for other methods.
