# Supplementary material for: A divide-and-conquer approach based on deep learning for long RNA secondary structure prediction: Focus on pseudoknots identification
Source: PLoS One. 2025 Apr 25;20(4):e0314837. doi: 10.1371/journal.pone.0314837 (PMC12026937; doi:10.1371/journal.pone.0314837)
Supplement: Attachment — Submitted filename: Response_to_Reviewers_auresp_2.pdf [file pone.0314837.s003.pdf]

A divide-and-conquer approach based on deep learning for long RNA secondary structure prediction: focus on pseudoknots identification

Omnes Loïc, Angel Eric, Bartet Pierre, Radvanyi François, Tahi Fariza

We thank the reviewers for their valuable comments.
Answers are in blue.

Reviewer #1: This paper proposes a novel divide-and-conquer method, DivideFold, to address the challenges of predicting RNA secondary structures, particularly pseudoknots, in long sequences. DivideFold recursively partitions RNA sequences into smaller fragments, applies existing structure prediction models to each fragment, and reassembles the overall structure. The method demonstrates superior performance in pseudoknot prediction compared to existing models, with significant computational efficiency for long RNA sequences. Furthermore, the authors provide open access to the DivideFold source code and datasets, enhancing the reproducibility of the research.

Comment : (Sato et al., 2021) and many other papers have pointed out that it is not possible to detect overfitting using only sequence-wise cross-validation with tools such as CD-HIT. In fact, some of the papers you cited as examples of sequence-wise cross-validation in your rebuttal believe that sequence-wise cross-validation alone is not sufficient, and they also conduct evaluation using family-wise cross-validation. To give an example, E2Efold (Chen et al., ICLR 2020) was evaluated for accuracy using only sequence-wise cross-validation, and while it achieved good accuracy there, subsequent validation by several papers by other groups pointed out that it fell into severe overfitting (e.g. Sato et al., 2021; Foo et al., 2022). Therefore, the current situation, where accuracy is evaluated only by sequence-wise cross-validation, cannot be acceptable. If the evaluation using the bpRNAnew dataset that I requested is inappropriate due to the problem of sequence length, you should construct an appropriate dataset to conduct the family-wise cross-validation.

Answer : Following your comment, we attempted to conduct a family-wise analysis of the benchmarked tools. However, sequences longer than 500 nt were removed from the bpRNA-new dataset, making it unsuitable for our approach. To solve this, we design a novel family-wise test dataset, which we name the FW (family-wise) dataset. For this, we followed the procedure that was used to create bpRNA-new. We gathered RNAs from newly identified families in the latest available version of Rfam at the time of writing, Rfam 15.0, that were not yet introduced in Rfam 12.2 when bpRNA-1m was created, ensuring that the FW dataset only contains different families from the ones in our train dataset. Then, we checked for sequence similarity with CD-HIT-EST at a 80% similarity threshold. In contrast to bpRNA-new, the FW dataset is based on Rfam 15.0 rather than Rfam 14.2 and contains twice as many new unseen families. Furthermore, it contains sequences up to 951 nt long. Unfortunately, since the release of Rfam 12.2, no new families including sequences longer than 1,000 nt were discovered. This is a significant issue preventing us from properly studying the generalization capabilities of DivideFold to newly discovered RNA families, since DivideFold is better suited for RNAs longer than 1,000 nt. Nonetheless, we decide to conduct this analysis for RNA sequences between 500 and 1,000 nt, even though few sequences in the FW dataset are longer than 500 nt. The results can be seen on **pages 14 and 15**, and more detailed results are given in the supplementary file. The results show that the performance drops significantly for all benchmarked tools on the FW dataset, even for

non-trained methods, suggesting that predicting the structures and pseudoknots of RNAs from new families remains a tough challenge.
